# Quercetin attenuates pentylenetetrazol-induced seizures in chicks through antioxidant and anti-inflammatory pathways

**DOI:** 10.5455/javar.2025.l963

**Published:** 2025-09-23

**Authors:** Suleiman Dawood Suleiman, Jian Salam Hasan, Karwan Idrees Jarjees, Aziza Raof Haji

**Affiliations:** 1 Department of Theriogenology, Physiology, and Anatomy, College of Veterinary Medicine, University of Duhok, Duhok, Iraq; 2 Department of Pathology and Microbiology, College of Veterinary Medicine, University of Duhok, Duhok, Iraq

**Keywords:** Anticonvulsant, anti-inflammatory, antioxidant, chicks, Quercetin

## Abstract

**Objective:**

This study examines the neuroprotective and anticonvulsant effects of quercetin on pentylenetetrazol (PTZ)-induced convulsive seizures in chicks.

**Materials and Methods:**

Sixty Ross broiler chicks were randomly assigned to six groups: a negative control, a positive control treated with PTZ at 80 mg/kg, a diazepam-treated group (5 mg/kg), and three quercetin-treated groups receiving 50, 100, and 200 mg/kg orally for six consecutive days, respectively. Two hours after the final dose, PTZ was administered to groups 3-6 to induce seizures. The onset of convulsions and mortality rates were recorded over a period of 3 h. Brain tissue was collected to determine biochemical parameters, malondialdehyde (MDA), total antioxidant capacity (TAO-C), interleukin-1β (IL-1β), and tumor necrosis factor-α (*TNF-α*).

**Results:**

The delay in the onset of convulsions and survival improvement were found in quercetin pretreatment, in a dose-dependent manner. The highly significant survival was found at 200 mg/kg (*p <* 0.001), and moderately at 50 and 100 mg/kg (*p <* 0.05). MDA (*p <* 0.05) and *TNF-α* (*p <* 0.01) levels were significantly decreased at all doses. TAO-C levels were significantly elevated, while IL-1β levels declined at 200 mg/kg (*p <* 0.05).

**Conclusion:**

Quercetin pretreatment at 200 mg/kg showed antioxidant and anti-inflammatory potential against PTZ-induced convulsive seizure as a preventive therapy for epilepsy management.

## Introduction

Epileptic seizure is the most common neurological disease globally, affecting well over 50 million people [1]. It is characterized by recurrent episodes of involuntary muscle contractions that may be restricted to a specific region of the body (local) or to the entire body (generalized) [2,3]. The main pathophysiological mechanism associated with its development is the disturbance between the excitatory neurotransmitter glutamate and the inhibitory neurotransmitter GABA (γ-aminobutyric acid) in the brain [4]. The reduction in GABAergic activity and the elevation in glutamatergic activity contribute to increasing neuronal excitability [5].

Experimental animals, mice and rats, are most commonly used as seizure models to study the process of epileptogenesis, drug screening, and how inflammation and oxidative stress contribute to the seizure progression [6]. In addition, chicks have also been used as seizure models by using maximal electroshock and PTZ and showing similar responses, such as disturbances between excitatory and inhibitory neurotransmitters, oxidative damage, and neuroinflammation [7].

In recent years, the relation between seizures and oxidative stress and neuroinflammation has been extensively studied [6]. The seizures are characterized by excessive reactive oxygen species (ROS) production and reduced antioxidant defenses in the brain [8]. The oxidative stress causes neuronal damage and promotes neuroinflammation in the brain, making it more susceptible to seizures [9]. The pro-inflammatory cytokine interleukin-1β (IL-1β), released during seizures, can disrupt the blood-brain barrier (BBB) and further infiltrate the inflammatory mediators into the brain [10]. The susceptibility to epileptic seizures is closely linked to BBB dysfunction [11], and seizure models are also observed with elevations in tumor necrosis factor-α (*TNF-α)*, BBB damage, and neuronal hyperexcitability [12,13].

The antiepileptic drugs used for epilepsy management often cause side effects, and about one-third of the patients with conventional antiepileptic drugs still have uncontrolled seizures [5]. This makes the need for new remedies that are safer and more effective for controlling the epileptic seizures [14,15]. Experimental studies have indicated that antioxidants can reduce the seizure-induced oxidative damage and neuroinflammation [16]. Quercetin has been demonstrated to have neuroprotective properties by reducing oxidative stress and inflammation in the brain [15,17].

Quercetin is a natural flavanol, widely distributed in various vegetables and fruits, and it is extensively studied for its antioxidant and anti-inflammatory properties in various pathological diseases [15,18]. It has been reported that quercetin can provide antioxidant and anti-inflammatory effects against kainic acid-induced seizures in mice by attenuating the production of IL-1β and *TNF-α* from glial cells [19]. Quercetin increased total antioxidant capacity (TAO-C) and reduced malondialdehyde (MDA) levels and decreased *TNF-α* and IL-1β in PTZ-induced seizures in the prefrontal cortex of mice [20] and attributed its antioxidant effects to the hydroxyl groups in its polyphenolic structure [3]. Quercetin at a lower dose of 25 mg/kg was found to reduce genetic absence seizures in rats by reducing *TNF-α* and *IL-6* levels, but it was not effective at higher doses (50 and 100 mg/kg) [21]. Furthermore, chronic intraperitoneal injection of quercetin could be used to exert anticonvulsant effects in rats subjected to penicillin-induced focal seizures [5]. The present study evaluates the anticonvulsant properties of quercetin in PTZ-induced seizures in chicks, focusing on its antioxidant and anti-inflammatory effects in brain tissue.

## Materials and Methods

### Ethical approval

The current study was performed with the approval of the Local Ethics Committee of the College of Veterinary Medicine, University of Duhok, Kurdistan Region, Iraq. The issue number was CVM2023/0310UoD.

### Laboratory animal

The present study was conducted on 60 Ross broiler chicks (one-day-old) of both sexes, which were purchased from Jeen Hatchery, Duhok, Iraq. The chicks were reared for 2 weeks before being used in this study in the poultry hall at a temperature of 28°C–32°C. They were maintained with constant lighting for locating feed and water *ad libitum* and reached the weight of 270–340 gm [22].

### Experimental design and induction of seizure

The chicks were divided into 6 groups of 10 chicks each. The first group, negative control (NC), received a subcutaneous (s.c.) injection of normal saline 1 ml/kg; the second group, positive control (PC), was injected with PTZ (Sigma, USA) 80 mg/kg s.c. [23]. The third group was treated with a single intramuscular injection of the standard anticonvulsant drug diazepam (ZEPADIC, Caspian Iran) at the dose of 5 mg/ml/kg [24]. The fourth, fifth, and sixth groups received quercetin 50, 100, and 200 mg/5 ml/kg, respectively, orally dissolved in saline with 1% DMSO for six consecutive days [14]. Two hours post-treatment in groups 3–6, the chicks received PTZ at the dose of 80 mg/kg, and then behavioral activities were observed and recorded using a digital camera for 30 min, and the mortality rate was recorded for 3 h [25].

### Collection of brain samples

At the end of the experiment, following 12 h of fasting, the animals were sacrificed by decapitation, and the brain was removed and stored in a plastic container at −20°C until use for biochemical analysis [25]. The 100 mg of brain tissue was minced and homogenized with 0.9 ml of cold phosphate buffer saline using an electrical homogenizer (Coyote, China) and centrifuged (DRAGONLAB, D3024R, China) at 5,000 rpm for 10 min at 4°C according to the kit manufacturer’s instructions. The supernatants were collected and stored at −20°C for biochemical analysis.

### Measurement of TAO-C and MDA

The TAO-C concentration in the tissue homogenate was determined using the ferric reducing antioxidant power method. The antioxidant substances in the homogenate reduced the ferric ions to the ferrous ions. The chromogenic reagent, tripyridyl triazine, was bound with ferrous ions and produced a blue-colored complex, which was measured at 593 nm using a microplate ELISA reader (BioTek, ELx808, USA). The TAO-C levels were calculated from a standard curve and expressed as µmol/gm of brain tissue [26].

The MDA levels in the tissue homogenate were quantified using a commercial sandwich ELISA kit (BT LAB, E0171Ch, China). The MDA bound to the captured antibody, and streptavidin-horseradish peroxidase (HRP) reacted with substrate to produce a blue color measured at 450 nm. MDA levels were calculated from the standard curve and expressed as nmol/gm of brain tissue.

### Measurement of IL-1β and TNF-α

The concentrations of inflammatory cytokines IL-1β and *TNF-α* in brain tissue homogenates were analyzed using commercially available competitive ELISA kits (BT LAB, EA00010Ch, China). The assay involved competition between the cytokine antigens and biotinylated antigens for binding to capture antibodies. Following sequential incubation with avidin-HRP and the chromogenic substrate, absorbance was determined at 450 nm. Cytokine concentrations, inversely proportional to color intensity, were determined using a standard curve and expressed as pg/gm of brain tissue.

### Statistical analysis

All biochemical data were analyzed using SPSS software (version 23). One-way analysis of variance was employed to compare the results between groups, and the values were expressed as mean ± SEM. Duncan’s post hoc test was used to identify significant differences at *p <* 0.05. Survival data were analyzed using Kaplan–Meier survival analysis, and differences in survival curves between groups were assessed using the log-rank (Mantel–Cox) test. Statistical significance was considered at *p <* 0.05 [27].

## Results

### Effects of quercetin on the onset of convulsions and mortality rates

**Figure 1.**
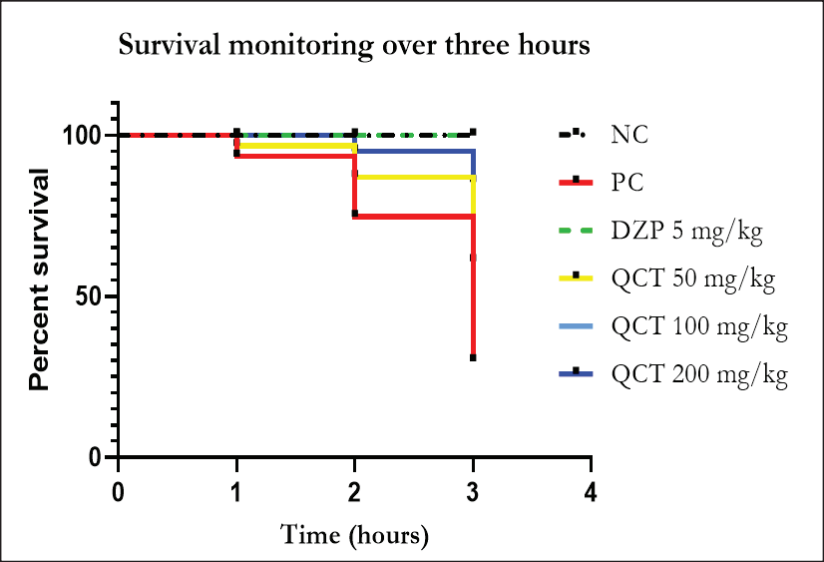
Kaplan–Meier survival curves showed the effects of different treatments on the survival of chicks over 3 h following PTZ-induced convulsions. NC, normal control; PC, positive control (PTZ 80 mg/kg); DZP, diazepam; QCT, quercetin.

The results presented in Table 1 demonstrate variations in the onset of convulsions and mortality rates across different treatment groups. The NC group, serving as the healthy controls, exhibited neither convulsions nor mortality. In contrast, animals in the PC group, which were treated with PTZ (80 mg/kg), showed convulsions within 5–25 min, with a mortality rate of 60%. In the group pretreated with diazepam (5 mg/kg), no convulsions or mortality were observed. In animals pretreated with quercetin (50 mg/kg), the onset of convulsion was delayed to 6–27 min, with the mortality rate reduced to 30%. A similar effect was observed with the 100 mg/kg dose, which slightly prolonged the onset to 7–27 min, and remained mortal at 30%. The onset of convulsion was further delayed at 200 mg/kg to 9–28 min, and reduced the mortality rate to 10%. The Kaplan-Meier survival curve (Fig. 1) showed a significant reduction in survival (*p <* 0.0001) in the PC group. Significant survival improvement was shown in quercetin at 50 and 100 mg/kg (*p <* 0.05) and at 200 mg/kg (*p <* 0.001).

**Table 1.** Effect of different treatments on the onset of convulsions and mortality in chicks, PTZ-induced convulsions.

Groups	Onset of convulsions (min)	Mortality rate (%)
Negative control	None	0
Positive control	5–25	60
Diazepam 5 mg/kg	None	0
Quercetin 50 mg/kg	6–27	30
Quercetin 100 mg/kg	7–27	30
Quercetin 200 mg/kg	9–28	10

### Effects of quercetin on oxidative stress and neuroinflammation biomarkers

The effects of quercetin on MDA, TAO-C, IL-1β, and *TNF-α* are shown in Table 2. The PTZ caused significant elevation (*p <* 0.001) in MDA levels and reduction in TAO-C levels (*p <* 0.01) in the PC group compared to the NC group. The group pretreated with diazepam showed that MDA levels significantly decreased (*p <* 0.001), while TAO-C levels increased (*p <* 0.05) compared to the PC group. Quercetin pretreatment at all doses resulted in a significant reduction in MDA levels (*p <* 0.01); however, only the highest dose (200 mg/kg) significantly increased TAO-C levels (*p <* 0.05) compared to the PC group.

Furthermore, IL-1β (*p <* 0.05) and *TNF-α* (*p <* 0.001) concentrations were significantly elevated in the PC group compared to the NC group. Diazepam pretreatment caused statistically significant reduced IL-1β (*p <* 0.05) and *TNF-α* (*p <* 0.001) levels relative to the PC group. Quercetin at 200 mg/kg significantly reduced IL-1β levels (*p <* 0.05), while all quercetin doses resulted in significantly lowered *TNF-α* levels (*p <* 0.01) compared to the PC group.

**Table 2.** Effects of quercetin on oxidative stress (MDA, TAO-C) and neuroinflammatory (IL1-β, *TNF-α*) markers in chicks with PTZ-induced convulsive seizures compared to control groups.

Groups	MDA nmol/gm	TAO-C µmol/gm	IL1-β pg/gm	*TNF*-α pg/gm
Negative Control	28.82 ± 1.67^B^	1.10 ± 0.10^A^	23.80 ± 0.89^B^	11.22 ± 1.12^C^
Positive Control	57.20 ± 2.78^A^	0.46 ± 0.05^D^	33.57 ± 2.88^A^	30.70 ± 3.10^A^
Diazepam 5 mg/kg	29.92 ± 2.52^B^	0.97 ± 0.13^AB^	24.30 ± 1.04^B^	14.58 ± 1.82^C^
Quercetin 50 mg/kg	38.07 ± 3.73^B^	0.58 ± 0.14^CD^	29.18 ± 1.58^AB^	22.17 ± 2.58^B^
Quercetin 100 mg/kg	37.07 ± 3.03^B^	0.66 ± 0.11^BCD^	27.75 ± 2.12^AB^	17.92 ± 1.73^BC^
Quercetin 200 mg/kg	36.60 ± 4.18^B^	0.88 ± 0.11^ABC^	26.70 ± 3.19^B^	17.11 ± 2.01^BC^

Values are expressed as mean ± SE. Different superscript letters within the same column indicate significant differences at *p* < 0.05, as determined by Duncan’s test; values sharing the same superscript letter are not significantly different.

When comparing the diazepam-treated group to the three quercetin-treated groups, only the highest dose of quercetin (200 mg/kg) produced effects that were statistically comparable to diazepam in reducing MDA and IL-1β levels and enhancing TAO-C levels. In contrast, the lower doses of quercetin (50 and 100 mg/kg) showed significantly weaker effects, as they differed significantly from the diazepam group, particularly in TAO-C and IL-1β parameters.

## Discussion

Oxidative stress and inflammation are important interlinked mechanisms that play crucial roles in both physiological and pathological conditions [28]. Many studies and research have revealed that oxidative stress, inflammation, and disruptions in excitatory-inhibitory neurotransmission contribute significantly to seizure development and are integral to the pathophysiology of epilepsy [18,29]. These mechanisms collectively influence neuronal excitability and seizure susceptibility, suggesting the importance of targeted therapeutic interventions to modulate these processes effectively [18]. In this study, the PTZ model was used to assess the neuroprotective properties of quercetin in chicks. Quercetin protected against PTZ-induced convulsions, with higher doses demonstrating greater effectiveness by delaying the onset of convulsions and reducing mortality. Although quercetin exhibited an apparent dose-dependent effect in improving survival, the 100 mg/kg dose did not further reduce mortality compared to 50 mg/kg, suggesting a possible plateau in its protective efficacy. This observation is supported by Abdulsahib and Al-Radeef [14], who reported that in a pilocarpine-induced epilepsy model in mice, both 150 and 200 mg/kg doses of quercetin reduced mortality by 33%, with no additional benefit observed at the higher dose. Studies in poultry showed that oral administration of quercetin at 200 mg/kg alleviated inflammatory responses in broiler chickens [30]. Furthermore, it was considered safe, with no clinical signs of toxicity observed at a dose of 2,000 mg/kg in broiler birds [31].

The biomolecular mechanisms involved in epileptogenesis include neurotransmitter dysregulation, ion channel alteration, receptor dysfunction, oxidative stress, and neuroinflammation pathways [32]. Oxidative stress serves as a key factor before and after seizures, leading to cellular damage, mitochondrial impairment, and disruption of antioxidant defenses [6]. The present findings showed that PTZ causes a significant increase in MDA levels and a reduction in TAO-C levels, indicating its oxidative damage in the brain tissue. A previous study showed that PTZ leads to oxidative damage in chicks by decreasing antioxidant enzymes catalase and glutathione peroxidase and increasing lipid peroxidation markers MDA and 8-isoprostane [4]. Previous studies have indicated that rats subjected to PTZ kindling exhibit an increase in MDA levels and a decrease in superoxide dismutase in the brain tissue [33]. The brain tissue is susceptible to oxidative damage because of its high oxygen consumption and richness in polyunsaturated fatty acids [6].

The current results showed that quercetin attenuates the oxidative stress induced by PTZ by decreasing MDA levels at all doses and increasing TAO-C levels at 200 mg/kg, exhibiting pronounced antioxidant effects similar to those of diazepam. Tavakoli et al. [20] reported that quercetin increases seizure threshold, reduces the MDA levels, and increases TAO-C levels in the prefrontal cortex in rats with PTZ-induced seizures. Quercetin has been shown to penetrate the BBB and exert neuroprotective properties against several brain disorders, including epileptic seizures [3]. The evidence supported the role of quercetin in various diseases through its antioxidant, anti-inflammatory, and neuroprotective effects [15]. The hydroxyl group in the polyphenolic structure of quercetin ameliorates the oxidative damage and scavenges the free radicals by inducing glutathione levels [3].

Quercetin has been shown to reduce microglial activation and *TNF-α* and IL-1β levels and increase the expression of anti-inflammatory cytokines in mice glial cells with induced seizures [34]. It exhibits anticonvulsant effects against penicillin-induced focal seizures in rats [5] and anti-inflammatory effects against lipopolysaccharide-induced inflammation in alveolar epithelial cells [35]. It has been reported that it exerts protective effects against diquat-induced cell death, scavenges ROS, and induces glutathione levels [36].

A significant inflammatory reaction was observed following PTZ-induced seizures, as indicated by the increased IL-1β and *TNF-α* levels in the PC group. In animal models of seizure, chemoconvulsants, such as PTZ, have been shown to elevate and upregulate pro-inflammatory cytokines through microglial activation in the brain [37]. Neuroinflammatory reactions are activated in a subset of central nervous system (CNS) disorders that are driven by neural-specific autoantibodies and linked to seizures [38]. Experimental research demonstrated that administration or overexpression of IL-1β increased seizure susceptibility in experimental animal models [14]. IL-1β can enhance excitotoxicity, the process in which high activation of glutamate receptors results in neuronal damage [10]. Excitotoxicity can influence acute seizures and the development of epilepsy; IL-1β potentially worsens this process by promoting glutamate release and modifying synaptic plasticity [39]. Conversely, inhibiting IL-1β signaling pathways can lessen both the intensity and frequency of seizures, indicating that IL-1β may directly contribute to the development of epilepsy and the initiation of seizures [40].

*TNF-α* is a multifunctional cytokine that has a debated contribution to epileptogenesis, as it appears to promote seizure development and trigger neuromodulatory effects [13]. As a key mediator in both inflammatory and neuromodulatory pathways, *TNF-α* has been considered a potential pharmacological target for various neurological disorders [41]. Given the conflicting findings, targeting *TNF-α* for treating CNS disorders seems premature [13]. In the present study, quercetin treatment significantly lowered the amounts of IL-1β and *TNF-α*, with a 200 mg/kg dose producing the most pronounced decrease. The weaker effect observed at 50 mg/kg may reflect subthreshold bioavailability for *TNF-α* suppression, possibly due to dose-dependent modulation of NF-κB signaling and limited cellular antioxidant capacity [18].

This finding aligns with Bakre et al. [42], who demonstrated that quercetin significantly reduced *TNF-α* levels compared to animals exposed to PTZ alone. The *TNF-α* suppression observed across all doses contrasts with findings from rodent-based studies, where lower doses were required to achieve anti-inflammatory effects [43]. This variation may be attributed to differences in species sensitivity, absorption kinetics in chicks, and the oral administration route, which could result in more gradual and sustained bioavailability of quercetin [30].

Quercetin’s anti-inflammatory properties suggest its potential neuroprotective role in epilepsy, likely by mitigating seizure-induced inflammation [42]. Evidence suggests that it exerts antiepileptic effects by lowering proinflammatory cytokine levels such as *TNF-α* in animals with kainic acid-induced epilepsy [19]. One study reported that serum and hippocampal cytokine concentrations were significantly elevated following seizure induction in rats but were markedly reduced by quercetin treatment, suggesting suppression of the proinflammatory response [18]. Tavakoli et al. [20] and Wu et al. [18] demonstrated its anti-inflammatory effects and a significant reduction in seizure susceptibility through decreased IL-1β and *TNF-α* gene expression in the brain during seizure induction in mice. Additionally, Xie et al. [44] and Alharbi et al. [15] stated that quercetin reduced inflammatory cytokine levels, including IL-1β release. In contrast, Abdulsahib and Al-Radeef [14] observed that quercetin did not have a statistically significant influence on reducing IL-1β levels in the plasma of pilocarpine-treated mice. Another study found that a 25 mg/kg dose of quercetin reduced the absence seizures by lowering *TNF-α* levels, whereas 50 and 100 mg/kg doses had no significant effect on *TNF-α* concentrations in rat brain tissue [21]. Adeoluwa et al. [42] revealed that pretreatment with quercetin at 12.5 and 25 mg/kg suppressed PTZ-induced seizures in mice by significantly reducing interferon-γ and IL-12 levels in the brain.

Conversely, Wu et al. [19] reported that treatment with 100 mg/kg quercetin significantly lowered IL-1β and *TNF-α* levels in microglial cells of mice treated with kainic acid. The enhanced anti-inflammatory efficacy of quercetin at all doses in chicks, compared to its limited effectiveness at higher doses in mice and rats, may be attributed to species-specific differences in absorption, metabolism, distribution, and BBB function. Supporting this, Sun et al. [30] reported that quercetin at 200 mg/kg effectively reduced lipopolysaccharide-induced inflammation and apoptosis in broilers by downregulating *TNF-α*, IL-1β, *IL-6*, and *IL-8*. Overall, these findings highlight quercetin as a protective therapy for reducing seizure-related neuroinflammation.

In neurological disorders such as epilepsy, the BBB serves as a major obstacle that restricts the effective delivery of therapeutics to the CNS. Therefore, when selecting pharmaceuticals for epilepsy treatment, the compound’s ability to efficiently cross the BBB is a critical consideration [45]. Several *in vivo *and *in vitro *studies have indicated that quercetin can cross the BBB after systemic administration and exert neuroprotective effects against various neurological disease conditions [18,46,47]. Hence, the ability of flavonoids, including quercetin, to cross the BBB supports their potential as viable candidates for preventing and managing neurological diseases, including epilepsy.

## Conclusion

No experiments have reported the neuroprotective role of quercetin on PTZ-induced epilepsy in chicks. Quercetin effectively suppressed seizures, reduced brain levels of MDA, IL-1β, and *TNF-α*, and increased TAO-C, indicating its antioxidant and anti-inflammatory activity. Among the tested doses, 200 mg/kg was the most effective in attenuating seizure severity and modulating oxidative and inflammatory markers, suggesting it may represent an optimal dose in this model. These observations highlight quercetin’s promise as a protective agent for epilepsy prevention. However, a comprehensive understanding of the molecular mechanisms underlying quercetin’s antiepileptic and neuroprotective functions remains essential for the advancement of novel therapeutic strategies for the management of epilepsy. Although quercetin has shown potential in preclinical studies, clinical evidence regarding its safety and mechanism of action remains limited. Quercetin may serve as a potential adjunctive protective agent for epilepsy; however, further investigation is needed to confirm its protective efficacy, including histopathological analysis of brain tissue to confirm neuroprotection at the structural level.

## List of Abbreviations

BBB, blood-brain barrier; CNS, central nervous system; C, degree Celsius; DMSO, Dimethyl sulfoxide; DZP, diazepam; ELISA, enzyme-linked immunosorbent assay; GABA, gamma-aminobutyric acid; gm, gram; HRP, streptavidin-horseradish peroxidase; IL-1β, interleukin-1 beta; kg, kilogram; MDA, malondialdehyde; ml, milliliter; mg, milligram; µmol, micromole; NC, negative control; nm, nanometer; nmol, nanomoles; PBS, phosphate buffered saline; PC, positive control; PTZ, pentylenetetrazol; pg, picogram; QCT, quercetin; ROS, reactive oxygen species; rpm, rotation per minute; SPSS, statistical package for the social sciences; SEM, standard error of the mean; s.c., subcutaneous; *TNF-**α*, tumor necrosis factor-alpha; TAO-C, total antioxidant capacity.
